# Framework for interprofessional case conferences – empirically sound and competence-oriented communication concept for interprofessional teaching

**DOI:** 10.3205/zma001461

**Published:** 2021-02-15

**Authors:** André Posenau, Marietta Handgraaf

**Affiliations:** 1 Hsg Bochum – Hochschule für Gesundheit, Bochum, Germany

**Keywords:** IPE, case conferences, communication, ICF, IPP

## Abstract

**Aims/objectives:** Interprofessional case conferences support future team-based approaches to healthcare, and inevitably require targeted communication between the various participants. However, the success of communication during a case conference must be learnt explicitly. The subject of conversation is often the only outcome of the case conference that is discussed in plenary or small groups. Communication processes are hardly taken into account. However, integrating process orientation and making communication relevant to goal achievement is mandatory in order to teach in a competence-oriented fashion in this area. The aim of this article is to present an empirically sound framework for teaching case conferences, with the help of which conversation processes can be practiced, evaluated and analysed in interprofessional case conferences.

**Methodology: **With the aid of literature analysis, insights from empirical conversation research and the International Classification of Functioning and Health (ICF), we have developed an empirically and theoretically sound framework for interprofessional case conferences. This is intended to support the training of communication skills and to serve as a basis for assessing them.

**Results: **In practice, it has been shown that embedding case conferences in higher education curricula is feasible and effective for a group size of 200 students. The framework has proven itself in verbal training while aligning itself with concepts of sharing for the negotiation of leadership, goals and decisions. In addition, it could also be used as a theoretical construct for the “interprofessional objective structured clinical examination” (iOSCE) at graduation from the module “Interprofessional Case Conference” at the Hochschule für Gesundheit.

**Conclusion: **The topics of interprofessional practice (IPP) and communication are now the subject of curricula in the health professions, both nationally and internationally. In addition, various competence settings are available that can support didactic orientation. However, the authors believe that there are no concrete imperatives for competence-oriented implementation in teaching and examination. In the area of communication teaching, one can integrate empirically sound concepts instead of induction into degree course for the health professions, in order to provide a basis for the further development of communicative competence in this field.

## Zusammenfassung

**Zielsetzung: **Interprofessionelle Fallkonferenzen stützen zukünftige teambasierte Versorgungsansätze und benötigen unweigerlich eine zielführende Kommunikation zwischen den unterschiedlichen Akteuren. Das Gelingen der Kommunikation während einer Fallkonferenz muss allerdings explizit gelernt sein. Gegenstand der Bearbeitung ist häufig nur das Ergebnis der Fallkonferenz, welches im Plenum oder Kleingruppen diskutiert wird. Die kommunikativen Prozesse werden dabei kaum berücksichtigt. Jedoch ist die Integration der Prozessorientierung und der Relevanzmachung der Kommunikation zur Zielerreichung obligatorisch, um kompetenzorientiert in diesem Bereich zu lehren. Ziel dieses Beitrags ist die Darstellung eines empirisch fundierten Frameworks zum Lehren von Fallkonferenzen, mithilfe dessen Gesprächsprozesse in interprofessionellen Fallkonferenzen geübt, bewertet und analysiert werden können.

**Methodik: **Mithilfe einer Literaturanalyse, Erkenntnissen aus der empirischen Gesprächsforschung und dem International Classification of Functioning and Health (ICF) wurde das empirisch und theoretisch fundierte Framework für interprofessionelle Fallkonferenzen entwickelt, das bei der Schulung der kommunikativen Kompetenzen unterstützen soll und bei der Bewertung dessen als Grundlage dient.

**Ergebnisse: **In der Praxis hat sich gezeigt, dass die curriculare Einbettung von Fallkonferenzen im Hochschulkontext bei einer Gruppengröße von 200 Studierenden machbar und effektiv ist. Das Framework hat sich bewährt, innersprachliche Aufgaben zu schulen und sich gleichzeitig an Konzepten des Teilens (sharing) für das Aushandeln von Leadership, Zielen und Entscheidungen auszurichten. Darüber hinaus konnte es auch als theoretisches Konstrukt für die interprofessionelle objective structured clinical examination (iOSCE) zum Abschluss des Moduls „Interprofessionelle Fallkonferenz“ an der Hochschule für Gesundheit eingesetzt werden.

**Schlussfolgerung: **Die Themen interprofessionelle Praxis (IPP) und Kommunikation sind mittlerweile national wie international Gegenstand der Curricula von Gesundheitsberufen. Darüber hinaus stehen verschiedene Kompetenzrahmen zur Verfügung, die bei der didaktischen Ausrichtung unterstützen können. Konkrete Imperative für die kompetenzorientierte Umsetzung in Lehre und Prüfungen fehlen jedoch nach Ansicht der Autoren. Anstelle der Induktion ist es auch im Bereich der Kommunikationslehre möglich, empirisch fundierte Konzepte in das Studium der Gesundheitsberufe zu integrieren, um eine Weiterentwicklung der kommunikativen Kompetenzentwicklung in diesem Segment zu fundieren.

## 1. Introduction

The health professions face the challenge of having to integrate efficient structures for cooperation into everyday healthcare in order to address the increasing complexity of healthcare requirements [1]. The goals for chronic care management are changing; they are geared less towards healing, and more towards improving the patient's daily functioning and quality of life [2]. In order to achieve these goals, effective cooperation between the various healthcare professions is particularly important in the care of chronic patients [3]. Interprofessional collaboration or teamwork is seen as a dynamic process that involves two or more healthcare professionals, sometimes with complementary backgrounds and skills. They pursue common health objectives in order to provide personalised care. Their cooperation is characterised by structured exchange, open communication and shared decision-making [4]. The WHO has been promoting such healthcare structures since 2010, but many challenges still lie ahead in practice [1]. Various barriers have been identified in the relevant literature, such as working towards healthcare objectives, ambiguities in the allocation of responsibilities, and the lack of continuity in the healthcare teams [5], [6]. In particular, the exchange of information across occupational boundaries requires knowledge of the roles of the other professions. The better this exchange functions, the fewer the team structures that are characterised by hierarchical relationships [5], [7], [8]. Effective communication in interprofessional healthcare interactions, based on common or shared visions and mutual respect, helps to strengthen cooperation in teams and the coordination of treatment [5], [9], [10]; this will ultimately implement more efficient care for people who have to live with chronic complaints [3]. In addition to simulating handovers and ward rounds, an interprofessional case conference appears to be an appropriate educational measure to increase knowledge and awareness of the role of other healthcare professionals in patient care [11], to promote specific communication strategies for negotiating objectives and decisions, and to develop interprofessional care plans on this basis [12]. These topics are hardly the subject of scientific conversation in German-speaking countries from a didactic perspective in terms of communication teaching [13], although many projects in this area have been stimulated by funding opportunities [14]. Conceptual development, empirical foundation of the concept and their subsequent implementation are therefore the focus of this contribution.

## 2. Interprofessional case conferences in an educational context

Germany. Existing training structures therefore provide few structural opportunities for interprofessional communication. They hinder the sharing of expertise between professions and better understanding of the roles of various potential team members [6], [8]. Interprofessional case conferences are a potential means of training in collaborative healthcare approaches. The prerequisites for success are that the participants assess the expertise of the other professions, have a clear understanding of their roles, and can access a framework for the organisation of case conferences [15]. In order to carry out an efficient and effective case conference, it is therefore essential to create targeted communication with each other and an appreciative culture of cooperation. Communication should be initiated by planned conversation instead of chance meetings in the corridor or only when problems occur, as is often the case in routine healthcare [15]. Person-centred approaches to healthcare require that information regarding personal needs and current health needs are discussed in order to negotiate decisions and common objectives [16], [17]. Only then will it be possible to develop a strategic plan that takes into account current health issues as well as future health needs [17]. The definition of Bell et al. [18] is used for the case conference: “A case conference is a multidisciplinary meeting of two or more health professionals to plan care for a specific person with chronic and complex care needs”.

The skills necessary for the conduct of interprofessional case conferences are shown in table 1 (Tab. 1). The promotion of this expertise should be reflected in the training concept; the supporting environment is discussed in the next section.

All these skills must be taken into account in teaching, and this should be realised according to the subject matter in practice, e.g. by conducting a case conference, which must be supplemented following its completion by the communication behaviour factor in the reflection phase (see table 2 (Tab. 2)). This raises the following question: what have we learnt from this, and is it possible to describe it verbally? The following framework was developed for this purpose.

## 3. Structure of interprofessional case conferences

**Figure 1:**
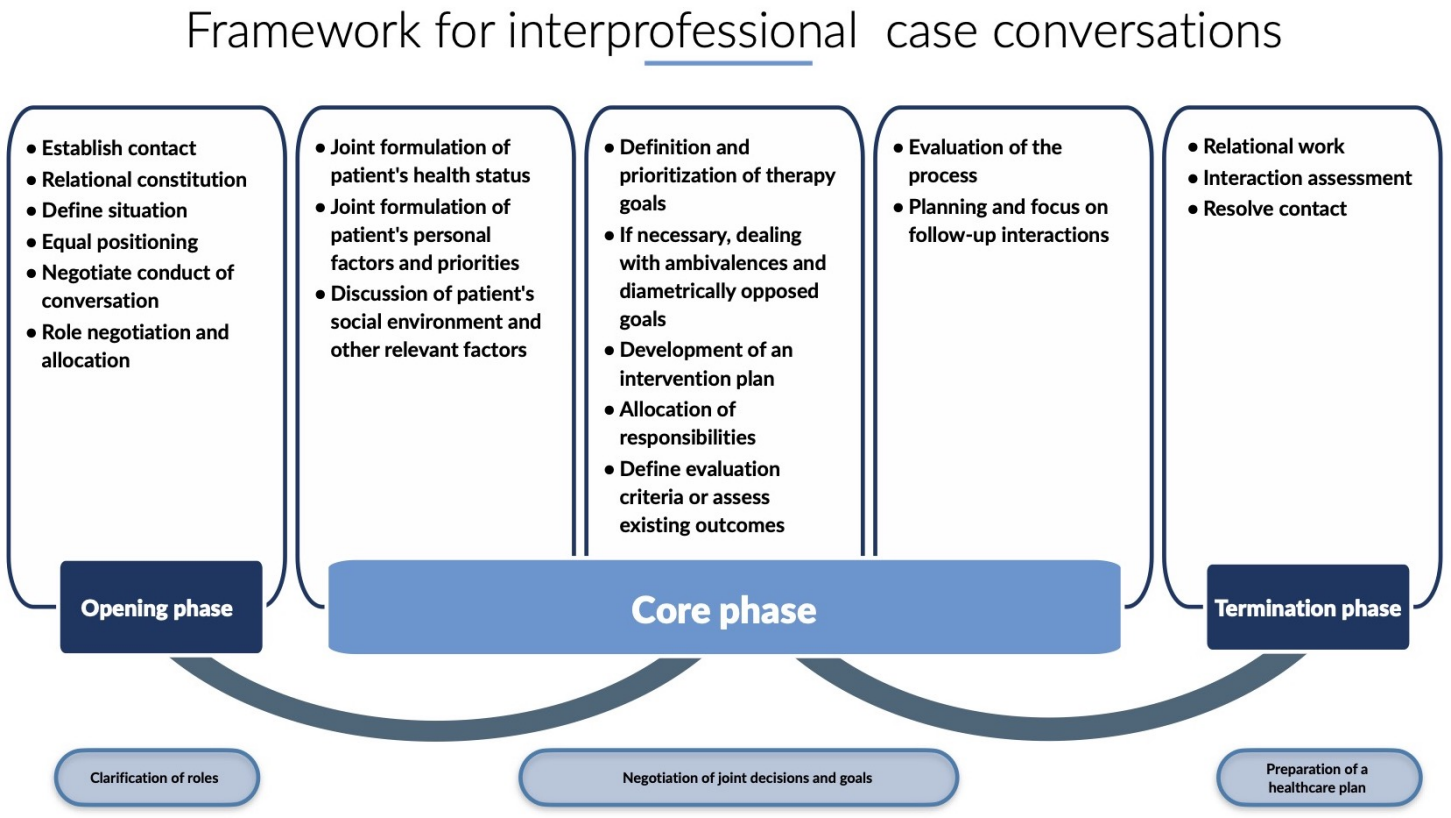
Structure of an interprofessional case conference (Posenau/Handgraaf, own presentation).

The implementation of interprofessional case conferences is a complex type of conversation based on a specific structure. This structure is oriented towards the tasks that need to be dealt with in the conversation. The processing of extra-linguistics tasks refers to concrete verbal actions that must be carried out if we are to classify the conversation as a case conference. We can describe a “prototype” of how an interprofessional case conference should be conducted. This ideal case was developed from several perspectives and serves as a framework for interprofessional case conferences (see figure 1 (Fig. 1)).

In developing the framework’s structure, the basic assumption from the perspective of communication analysis is that conversations are actively constituted in the form of interrelated processes. We must also take into account methodicity, in other words the cultural dimension, as well as pragmaticity [19]. The latter characteristic is particularly relevant in this context, since problem-solving by the participants is a central feature of the conversation. Here we distinguish between verbal and extra-linguistics tasks. In other words, everything that must be continually realised by all participants in each conversation (purely verbal) and everything that needs to be processed as a concrete task so that one can speak of a specific type of conversation. In this case, it is about the specifics of the conversation type of the interprofessional case conference. This inductive understanding of conversations makes it possible to grasp the type of conversation empirically with the aid of conversation analysis, and apply it in an application-oriented manner.

At the communicative level, the conversation during a case conference concentrates particularly on the control and active constitution of conversational phases, as well as coordination of the various conversational phases and internal language tasks described above [20]. Accordingly, conversations always consist of opening, core, and termination phases, which must be actively established by the parties involved. The opening and closing phases are primarily reserved for structural and relational issues. The core phase is significantly more complex, and it is possible to distinguish various types of conversation. For example, the issues raised in case conferences must be processed in a manner different from that for a medical history [20].

Deppermann [19] follows Kallmeyer in this sense in speaking of different levels of reality, which must be actively constituted by the participants at every moment of the conversation. There is therefore always a task for the participants at the micro-level:

- to organise the conversation,
- to formulate the issues,
- to coordinate action at the micro-level,
- to establish and maintain social relationships and identity,
- to constitute the modality of the interaction,
- and to generate reciprocity.

These verbal tasks are present in every conversation, and an inductive perspective helps when observing interactions and makes it empirically possible to analyse and describe them.

At this level, classical methods of communication become relevant, although they have so far been regarded as the sole domain of communication teaching. For example, some texts refer to techniques [21] such as questioning techniques or techniques for conducting conversations, or even more complex procedures such as active listening.

At the micro-level, conversation analysis can therefore be a basis for empirical foundation with its diverse options for analysis and description, without which the fluid object of investigation could not be grasped at all. At the macro-level, the model of Packard et al. [22] is used on the basis of the ICF [23] to identify the extra-linguistics tasks, as outlined below.

At the action-oriented level, the interprofessional structure of case conferences deals with extra-linguistics issues. This refers to aspects of conversation that are decisive and substantive in terms of the content of a case conference. If the appropriate tasks have not been processed, one cannot speak of a case conference. Three essential criteria were identified in the literature for this purpose [23], [22], [24]. Firstly, participants in a case conference should speak a language that is understandable to all and be person-centred from a biopsychosocial point of view. The International Classification of Functionality, Disability and Health (ICF) [23] was used as a theoretical model for this purpose. Packard et al. [22] developed an interprofessional, team-based decision-making structure based on the ICF, which enables diverse tasks in the interaction context to be managed in case processing. The advantage of this model is the transfer and integration option across all professions.

This structure serves two central levels that are relevant when conducting a case conference:

1. a global perspective of the course of the conversation and the resulting tasks for the participants from a communicative viewpoint; and
2. an action-oriented perspective from which the best possible interprofessional care is explored in a person-centred manner.

The authors speak of competence in this context if the ability to develop case conferences is observable and tangible, i.e. the ability to process tasks and manage the process is empirically apparent. With particular reference to case conferences, this means that students learn to work communicatively on the following case-based tasks:

On the basis of Packard et al. [22], further mandatory extra-linguistics tasks were subsequently identified. All processes related to cooperation now focus on these tasks [24]. The authors regard cooperation in teams as characterised by situational and cross-sectoral activity with regard to a specific healthcare situation. This necessarily leads to a cultural change in the health system [17]. Secondly, this is based on the concept of sharing [22], [25]: shared practice team leadership [26], shared goal-setting [17], [27], [28] and shared decision-making [17], [27], [28]. According to the literature, the leadership function in case conferences is assigned to the profession that has the greatest expertise in the health issues of the patients, and can therefore assume the leadership and responsibility for the sub-processes and the outcome. This leadership role must be renegotiated at each case conference. Targeting and joint decision-making accelerate coordinated negotiation in the case conference, while the needs of the patients guide the procedure. The various approaches of the diverse professions are made apparent by exchanging information. In the case conference, information exchange will help to decide which option(s), i.e. objectives and decisions, need to be prioritised. Thirdly, an inter-professional healthcare plan is drawn up on this basis. It is developed by all participants [12], [29] and should be aligned with current evidence [22], [29].

These communicative and action-related process levels were adapted and integrated into the framework of the interprofessional case conference (see figure 1 (Fig. 1)). This framework should be regarded as the basis for training in the case conferences. Its implementation in teaching is exemplified by the module “Interprofessional Case Conferences” at the Hochschule für Gesundheit. The students thus learn to process the tasks represented in the example. The framework serves as an orientation to the concept of the teaching content and for reflection, in order to become more precise in the description and evaluation of communication processes.

## 4. Framework conditions at the Hochschule für Gesundheit Bochum

In practice, the case conference will be held with students in midwifery, nursing, physiotherapy, occupational therapy and speech therapy. A group of 20 interprofessional students will come together each week for ten weeks, making a total of about 200 students in the course of one semester. A “case conference” teaching session has a clear structure, as shown below (see table 2 (Tab. 2)). The case conferences take place in the 7^th^ semester and have a workload of three credit points. Due to their time slot in the 7^th^ semester, students have usually passed (or are about to pass) their final state examination. Students from two or three professions work together during a case conference.

Unlike traditional case conferences, the authors of this concept go beyond the development and analysis of the best possible healthcare outcome by integrating reflection regarding the communication processes into the teaching units. It involves not only “What do we do?”, but also “How do we do it?”.

## 5. Consequences for communication theory in practice

As a didactic starting point in the module, the framework and its theoretical foundation also have consequences for communication theory and examination methodology. First and foremost, communication based on these assumptions must be taught as a process-oriented procedure. The transfer of knowledge about phases, processes and tasks is essential; pure orientation to skills, e.g. communication techniques, is not enough. The processing of verbal and extra-linguistics tasks (oriented towards the ICF, cooperative decision-making and goal-setting as well as negotiation of leadership) is key; the processing of topics alone is inadequate. At the same time, the framework supports the conversation of professional boundaries. It is inevitable that insights into the working methods of other professions are gained, and that new strategies are identified and negotiated. However, teaching communication must bring diverse professions together, which is quite a challenge when finding the right place for it in the curriculum [30]. However, online formats can be used and spatial barriers can be overcome.

There is a further consequence of the test. Given the basic assumption that *“assessment drives learning”* [31], a competence-oriented examination is necessary at the end of the module. Until now, teaching has concentrated on cases at the expense of communication development. Linking these two aspects will make the contact time more efficient and effective. The framework makes it possible to observe the process more precisely and evaluate it at the same time, because each of the aspects discussed above can be captured empirically. This means that the evaluation process has an empirical basis, instead of being supported by introspection on the part of the evaluators. This is very relevant, since it is not satisfactory to base the evaluation exclusively on the outcome of the case conferences. If students need to deal with the complex requirements assigned to them in practice by interprofessional interactions, the most relevant criterion is performance. It is becoming apparent that case conferences can be a suitable method for achieving this aim.

## Profiles

**Name of the location: **University of Applied Sciences for Health, Bochum

**Subject/professional group: **Bachelor's degree programs for the professional groups of nursing, physiotherapy, occupational therapy, speech therapy and midwifery.

**Number of learners per year or semester:** a total of approximately 250 students per year.

**Has a longitudinal communication curriculum been implemented?** Yes, since the winter semester 2016.

**In which semesters are communication and social skills taught?** For all majors in the interprofessional modules in 2^nd^, 3^rd^, 6^th^ and 7^th^ semesters, as well as in the subject-specific modules.

**What teaching formats are used?** Lectures, practical exercises in skills labs, project week, and case conferences. 

**In which semesters are communication and social skills tested?** As module exams in the 3^rd^ and 7^th^ semester, which are graded.

**Which examination formats are used? **In the 3^rd^ semester a written exam, in the 7^th^ semester an interprofessional objective structured clinical examination (iOSCE).

**Who is responsible for the development and implementation? **The module leaders and two representatives from each of the five study areas of the School of Health develop and agree on the examination formats in the IPE committee. 

## Current professional roles of the authors

- Prof. Dr. phil. André Posenau: Professor for Interaction and Interprofessional Communication Nursing and Health Professions at the Department of Nursing Science at the Hochschule für Gesundheit (hsg) in Bochum. Main research interests: interprofessional communication and interprofessional didactics, empirical communication and social research, eHealth
- Dipl. Med.-päd Marietta Handgraaf: research associate in the department of physiotherapy at the Hochschule für Gesundheit (hsg) in Bochum. Main fields of work: Curricular developments and development of examination formats, interprofessional learning and action, professional policy issues.

## Competing interests

The authors declare that they have no competing interests. 

## Figures and Tables

**Table 1:**
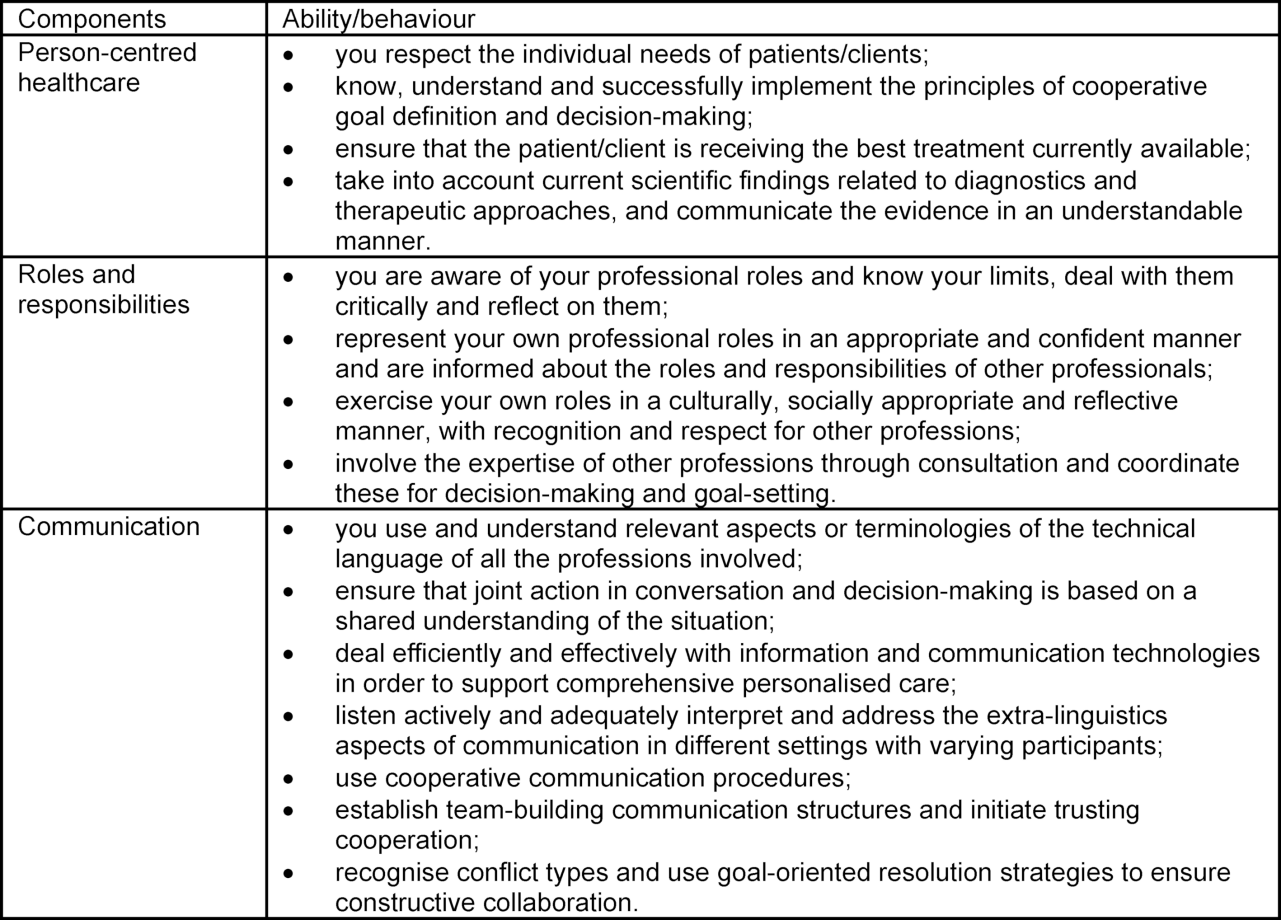
Overview of expertise development in the interprofessional case conference

**Table 2:**
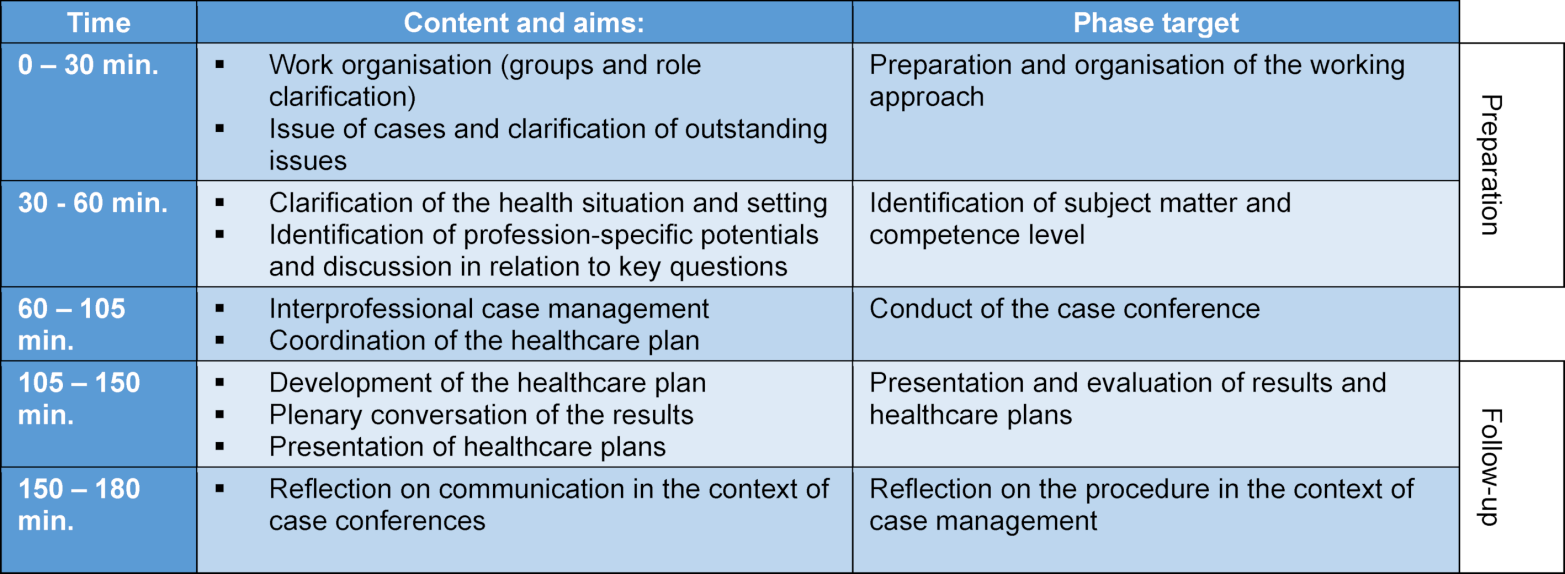
Schedule for a teaching unit (Posenau/Handgraaf, own presentation).
